# Predictors of Higher Peak Exercise Oxygen Uptake in a Cohort of Adult Patients with Fontan Circulation

**DOI:** 10.3390/jcm15103805

**Published:** 2026-05-15

**Authors:** Andrzej Wittczak, Mateusz Kobierecki, Maciej Banach, Agata Bielecka-Dabrowa

**Affiliations:** 1 Department of Preventive Cardiology and Lipidology, Medical University of Lodz, 90-419 Lodz, Poland; maciej.banach@umed.lodz.pl; 2 Department of Cardiology and Congenital Diseases of Adults, Polish Mother’s Memorial Hospital Research Institute, 93-338 Lodz, Poland; 3 Department of Diagnostic Imaging, Polish Mother’s Memorial Hospital Research Institute, 93-338 Lodz, Poland

**Keywords:** adult congenital heart disease, Fontan circulation, univentricular heart, predictors, cardiopulmonary exercise testing

## Abstract

**Background/Objectives**: Percent achieved of predicted peak exercise oxygen uptake (%VO_2pred_) is a prognostic factor for patients with Fontan circulation. The main purpose of this study was to determine predictors of higher %VO_2pred_ in a cohort of adult Fontan patients. **Methods**: Medical records of 50 adult Fontan patients who underwent cardiopulmonary exercise testing were reviewed. All patients were divided into two groups according to the mean value of %VO_2pred_ and, in separate analysis, according to the morphology of the systemic ventricle. Spearman’s rank correlation was used to examine the relationship between %VO_2pred_ and blood biomarkers. Regression analyses were used to identify predictors of %VO_2pred_. **Results**: The median age of all patients was 22 years, and 50% were female. The systemic ventricle was dominant right in 23 patients. Negative correlations were found between %VO_2pred_ and N-terminal prohormone of brain natriuretic peptide, mean cell hemoglobin concentration, and ferritin, and positive correlations were found between %VO_2pred_ and total protein, total iron-binding capacity. Higher chronotropic index [CI] (β = 0.31; *p* = 0.009), higher maximal diastolic blood pressure during CPET [DBP_max_] (β = 0.4; *p* = 0.001), and lower serum concentration of high-sensitivity troponin T [hsTnT] (β = −0.31; *p* = 0.007) were significantly and independently associated with %VO_2pred_. **Conclusions**: Higher CI and DBP_max_ and lower hsTnT were identified as independent predictors of %VO_2pred_ in this cohort. These findings suggest that the absence of chronotropic incompetence is a positive predictor of exercise capacity. Furthermore, hsTnT shows potential as a useful biomarker in this population. Further studies are needed to validate these parameters for clinical assessment and risk stratification in Fontan patients.

## 1. Introduction

The Fontan operation represents the definitive surgical intervention for appropriately selected patients with complex congenital heart defects involving a single functional ventricle [1]. The procedure isolates the pulmonary and systemic circulations by routing deoxygenated venous blood directly to the lungs for passive oxygenation, reserving the single functional ventricle exclusively for systemic output [2].

Exercise testing plays a crucial role in the adult congenital heart disease (ACHD) population because assessing quality of life and functional capacity is essential for evaluating the effectiveness of intervention [1]. Cardiopulmonary exercise testing (CPET) is particularly useful for patients with ACHD, as it provides a more comprehensive evaluation of functional capacity and physical fitness. Additionally, its endpoints correlate well with morbidity and mortality [1,3]. Therefore, the European Society of Cardiology (ESC) guidelines recommend that serial exercise testing should be included in long-term follow-up protocols [1].

Among the parameters obtained in CPET, oxygen uptake (VO_2_), is one of the most important. VO_2_ during physical activity is a measure of aerobic capacity [4]. The values of VO_2max_ or (more commonly) VO_2peak_ are a key focus when interpreting CPET results. VO_2max_ represents the amount of oxygen uptake during maximum physical effort and, therefore, can be achieved only in healthy people who can perform maximum exertion. For individuals with cardiovascular or respiratory conditions, reaching VO_2max_ is often unachievable or unsafe. Therefore, VO_2peak_, which reflects oxygen uptake at the peak of exercise, is used as an alternative [4]. Importantly, in order to reduce interindividual variations, VO_2peak_ should be expressed as percent achieved of predicted peak exercise oxygen uptake (%VO_2pred_). It is recommended to use the Wasserman and Hansen equation to calculate the predicted oxygen uptake value [4,5]. Peak oxygen uptake is the primary indicator of exercise capacity and is highly predictive of outcomes in patients with congenital heart disease [6]. Patients with Fontan circulation typically have reduced %VO_2pred_, which averages between 60 and 65% [6,7]. However, the observed range is wide, and a subgroup with normal %VO_2pred_ values exists (i.e., ≥80% predicted; sometimes called “super-Fontan”) [6].

A single functional ventricle supporting the Fontan circulation can have right, left, or indeterminate ventricular morphology, depending on the initial heart defect. Due to differences in morphology, it is generally believed that a single functional ventricle with right ventricular morphology is significantly less adapted to generating long-term systemic pressures than a single functional ventricle with left ventricular morphology [8]. Although data from older studies was inconclusive, recent publications proved that Fontan patients with a systemic right ventricle have a higher incidence of adverse cardiovascular events, including atrial arrhythmia, cardiac transplantation, and all-cause mortality [8].

Since %VO_2pred_ is a prognostic factor for patients with Fontan circulation, variables associated with oxygen uptake should be thoroughly studied. The main purpose of this study was to determine predictors of higher %VO_2pred_ in a cohort of adult patients with Fontan circulation.

Selected elements of this study were previously presented at the European Society of Cardiology Congress 2025 in Madrid [9] and at the 29th International Congress of the Polish Cardiac Society in Krakow [10].

## 2. Methods

### 2.1. Study Population

The retrospective cohort study included 50 patients with Fontan circulation who underwent CPET during elective hospital admission at the Department of Cardiology and Congenital Diseases of Adults, Polish Mother’s Memorial Hospital Research Institute in Lodz, Poland, between 2019 and 2024. The inclusion criteria were as follows: (1) age of 18 years or older; (2) presence of the complete Fontan circulation; (3) undergoing CPET during hospitalization.

In our department, CPET is routinely performed in all Fontan patients without medical contraindications. The main factors that disqualify patients from CPET are: (1) lack of consent to undergo the test; (2) patient’s inability to cooperate and/or give informed consent to undergo the test; (3) presence of symptoms of advanced heart failure with NYHA (New York Heart Association) IV severity of symptoms; (4) presence of significant arrhythmia; (5) presence of advanced respiratory failure; (5) presence of active systemic infection, including infective endocarditis; (6) documented myocarditis or pericarditis; (6) severe hyperthyroidism or hypothyroidism; (7) pregnancy or lactation; (8) having undergone surgery or major trauma within the past month.

The study was conducted with the approval of the Bioethics Committee at the Polish Mothers’ Memorial Hospital Research Institute (resolution No. 12/2024 of 20 February 2024). The aforementioned Bioethics Committee exempted the study from obtaining patient consent because it is a retrospective cohort analysis using de-identified data.

### 2.2. Data Gathered

#### 2.2.1. Medical Records

Data from the medical records of all included patients were collected. This data included general medical information (age, gender, weight, height), as well as data characterizing the Fontan circulation: signs and symptoms; blood oxygen levels at rest and during exertion; types of procedures performed and the ages at which they were performed; presence of fenestration or the age at which it was closed; type of functionally univentricular heart (initial defect)—including systemic ventricular morphology (left, right, or of indeterminate morphology); pharmacotherapy used; available cardiac catheterization data; and diagnoses of comorbidities (including complications specific to the Fontan circulation).

#### 2.2.2. Laboratory Tests

A standard set of laboratory tests was performed in all included patients during their hospitalization. Samples were collected after a minimum 12 h fasting period. The following tests are routinely performed in Fontan patients at our center: complete blood count, prothrombin time, activated partial thromboplastin time, serum glucose, alanine aminotransferase, aspartate transaminase, gamma-glutamyltransferase, bilirubin, C-reactive protein, creatinine, urea, sodium, potassium, calcium, total protein, albumin, thyroid-stimulating hormone, thyroxine, triiodothyronine, vitamin D, iron, total iron-binding capacity, unsaturated iron-binding capacity, ferritin, a lipid profile, the N-terminal fragment of brain natriuretic peptide (NT-proBNP), high-sensitivity troponin T (hsTnT), and alpha-fetoprotein. All analyses were conducted in the hospital’s central laboratory.

#### 2.2.3. Transthoracic Echocardiography

All patients included in the study underwent transthoracic echocardiography (TTE) using the Vivid E95 system (GE Healthcare, Chicago, IL, USA). Experienced ACHD specialists performed the examination with consideration of the nature of single-ventricle circulation [11].

#### 2.2.4. Cardiopulmonary Exercise Testing (CPET)

All patients underwent symptom-limited cardiopulmonary exercise testing (CPET) using a T2100 treadmill (GE Healthcare, Chicago, IL, USA) or an electromagnetically braked upright cycle ergometer (Bike M, Cortex Biophysik GmbH, Leipzig, Germany). A METALYZER 3B metabolic gas analyzer (CORTEX Biophysik GmbH, Leipzig, Germany), integrated with the MetaSoft Studio version 5.8.4 application software of CORTEX systems, was used. The examinations were performed by experienced physicians according to current standards [4]. Blood pressure was measured manually using the auscultatory method by experienced electrocardiography technicians.

Age-predicted maximal heart rate (HR_max_) was calculated using the standard equation: HR_max_= 220 − age. Heart rate reserve (HRr) was defined as the difference between HR_max_ and peak heart rate (HR_peak_) during exercise. Chronotropic index (CI) was defined as an index of maximal predicted HR reserve achieved and was accordingly calculated using following equation: CI= (HR_peak_ − resting HR)/[(220 − age) − resting HR)] [12].

The MetaSoft Studio software utilized the Wasserman and Hansen equation to calculate the %VO_2pred_ for all patients [5].

#### 2.2.5. Electrocardiography

All patients included in the study underwent 24 h Holter electrocardiography monitoring with the use of Schiller medilogAR or medilog FD12 Plus recorders (both Schiller AG, Baar, Switzerland). The recordings were analyzed by experienced cardiologists using medilog Darwin2 version 2.1 (Schiller AG, Baar, Switzerland) or CardioDay version 2.6 (GE HealthCare, Chicago, IL, USA) software.

#### 2.2.6. Ambulatory Blood Pressure Monitoring

All patients included in the study underwent ambulatory blood pressure monitoring (ABPM) with the use of Schiller BE-102 plus device (Schiller AG, Baar, Switzerland); the recordings were analyzed using medilog Darwin2 software version 2.1 (Schiller AG, Baar, Switzerland).

### 2.3. Statistical Analysis

The main data analysis was performed using the STATISTICA 13.1 software package (StatSoft/TIBCO Software Inc., Palo Alto, CA, USA). Regression analyses were performed using the SciPy package version 1.16.0 for the Python 3.12.13 programming language.

Statistical significance was defined as a *p*-value < 0.05. Shapiro–Wilk and Levene’s tests were utilized to evaluate data normality and the homogeneity of variances, respectively. Categorical variables are reported as frequencies and percentages. Continuous data are expressed as means and standard deviations for normally distributed variables or as medians with interquartile ranges (Q1–Q3) for non-normally distributed data. Between-group comparisons for continuous variables were conducted using Student’s *t*-test (for normally distributed data with equal variances) or the Mann–Whitney U test (for skewed data or unequal variances). Dichotomous variables were compared using the chi-squared test, applying Yates’ continuity correction or Fisher’s exact test where appropriate. Missing values were replaced via mean imputation.

All 50 patients were divided into two groups (both n = 25) according to the mean value of %VO_2pred_ (group A with %VO_2pred_ > 62.3% and group B with %VO_2pred_ ≤ 62.3%; %VO_2pred_ = 62.3% was a mean value for all patients; the values were normally distributed).

To compare patients with systemic ventricle of right and left ventricular morphology, separate analysis was performed, in which all patients were divided into two groups according to systemic ventricle (left = LV group, right = RV group, both n = 23; 4 patients had systemic ventricle of indeterminate morphology and were excluded from this sub-analysis).

Spearman’s rank correlation was used to examine the relationship between %VO_2pred_ and blood biomarkers. Correlations that were statistically significant were reported.

Simple linear regression analyses were used to investigate variables that were correlated with %VO_2pred_, and significant variables were incorporated into multivariable forward stepwise regression analysis.

## 3. Results

### 3.1. Cohort Data

The median age of all patients was 22 years (IQR 20–24), and 50% of them (n = 25) were female. The systemic ventricle was dominant left in 23 patients (46%), dominant right in 23 patients as well (46%), and of indeterminate morphology in four patients (8%).

The initial heart defects were as follows: A total of 21 (42%) patients with hypoplastic left heart syndrome (HLHS); 17 (34%) patients with tricuspid atresia; six (12%) patients with double inlet left ventricle (DILV); two (4%) patients with L-transposition of the great arteries (L-TGA); one (2%) patient with atrioventricular canal defect and TGA; one (2%) patient with TGA and pulmonary stenosis; and two (4%) patients with defects that were described as “common ventricle” (one with pulmonary artery stenosis, the other with total anomalous pulmonary venous connection (TAPVC)).

Out of 50 patients, 48 (96%) had the extracardiac conduit total cavopulmonary connection (TCPC) type of the Fontan operation; and the remaining 2 (4%) had the intra-atrial lateral tunnel TCPC type of the Fontan operation.

Baseline acid–base status was normal across the cohort.

Clinical characteristics of the study cohort are shown in Table 1.

### 3.2. Comparisons Between Group A (%VO_2pred_ > 62.3%) and Group B (%VO_2pred_ ≤ 62.3%)

The percent of female gender (64% vs. 36%, *p* = 0.047), resting oxygen saturation [94% (IQR 92–96) vs. 92% (IQR 91–95), *p* = 0.03], chronotropic index [77.78% (IQR 75–87.5) vs. 60.63% (IQR 47.24–78.45), *p* = 0.007], and oxygen uptake at anaerobic threshold [50% (IQR 44–56) vs. 43% (IQR 33–49), *p* = 0.03] were significantly higher in group A compared to group B. The use of beta-blockers (16% vs. 60%, *p* = 0.003), median number of ventricular extrasystolic beats [2 (IQR 0–17) vs. 35 (IQR 1–440), *p* = 0.03], median serum concentration of high-sensitivity troponin T [hsTnT] [4.7 pg/mL (IQR 3.6–5.5) vs. 5.7 pg/mL (IQR 4.4–6.7); *p* = 0.03], and median heart rate reserve [20 (IQR 12–30) vs. 50 (IQR 25–65), *p* = 0.004) were significantly lower in group A compared to group B.

Clinical characteristics of the study cohort according to the mean %VO_2pred_ are shown in Table 1.

### 3.3. Comparisons Between RV Group and LV Group

Angiotensin-converting-enzyme inhibitors (ACE-Is) and mineralocorticoid receptor antagonists (MRAs) were used significantly more frequently in the RV group than in the LV group (96% vs. 61%, *p* = 0.01; and 91% vs. 61%, *p* = 0.003, respectively). Pulmonary artery stenting (70% vs. 17%, *p* = 0.0008), median number of ventricular extrasystolic beats [ExV] [33 (IQR 2–440) vs. 2 (IQR 0–39), *p* = 0.04], mean activated partial thromboplastin time (34.1 s ± 4.89 vs. 31.14 s ± 3.34, *p* = 0.02), median serum concentration of thyroid stimulating hormone [3.62 uIU/mL (IQR 2.04–5.37) vs. 2.39 uIU/mL (IQR 1.74–3.19), *p* = 0.03], and median serum concentration of vitamin D [28.5 ng/mL (IQR 22.3–35.12) vs. 21.6 ng/mL (IQR 17.8–31.3), *p* = 0.03] were also significantly higher in the RV group compared to the LV group. Interestingly, median concentrations of N-terminal prohormone of brain natriuretic peptide [NT-proBNP] and high-sensitivity troponin T [hsTnT] were significantly lower in the LV group than in the RV group [65 pg/mL (IQR 26–145) vs. 139 pg/mL (IQR 54–391), *p* = 0.04; and 4.7 pg/mL (IQR 3.4–5.5) vs. 5.7 pg/mL (IQR 4.4–7.3), *p* = 0.03, respectively]. Mean %VO_2pred_ was lower in the RV group than in the LV group, but the difference was not significant [60.35% ± 12.19 vs. 66.09% ± 11.96, *p* = 0.11].

Clinical characteristics of the study cohort according to the systemic ventricle are shown in Table 2.

### 3.4. Correlations Between %VO_2pred_ and Biomarkers

Negative correlations were found between %VO_2pred_ and NT-proBNP (r = −0.29, *p* = 0.04), ExV (r = −0.31, *p* = 0.03), mean cell hemoglobin concentration [MCHC] (r = −0.31, *p* = 0.03), and ferritin (r = −0.29, *p* = 0.04). Total protein and total iron-binding capacity (TIBC) were positively correlated with %VO_2pred_ (r = 0.29, *p* = 0.04 for both).

### 3.5. Regression Analyses

Higher CI (β = 0.31; *p* = 0.009), higher maximal diastolic blood pressure during CPET [DBP_max_] (β = 0.4; *p* = 0.001), and lower hsTnT (β = −0.31; *p* = 0.007) were significantly and independently associated with %VO_2pred_ [R^2^= 0.433; adjusted R^2^ = 0.396].

The results of the regression analyses are shown in Table 3.

## 4. Discussion

Our study showed that patients with better %VO_2pred_ (defined as >62.3%—group A) had significantly higher chronotropic index, lower use of beta blockers, and lower mean heart rate reserve. Fontan patients with systemic RV had more ventricular arrhythmia and higher levels of NT-proBNP/hsTnT; however, no differences in parameters related to exercise capacity were statistically significant while comparing systemic RV and LV. Higher CI and DBP_max_ and lower hsTnT were identified as independent predictors of %VO_2pred_ in our cohort of adult Fontan patients.

Lower values of chronotropic index and higher values of heart rate reserve are characteristics of chronotropic incompetence. The term “chronotropic incompetence” can be defined as “the inability to increase the heart rate adequately during exercise to match cardiac output to metabolic demands” [13]. This condition is frequently observed in patients with Fontan circulation and is closely linked to reduced exercise capacity [14,15]. Indeed, we identified higher CI as an independent predictor of %VO_2pred_ in our cohort. This suggests that a more adequate HR response to exercise predicts better exercise capacity. However, it should be noted that in the Fontan circulation, chronotropic incompetence is currently considered to be secondary to hemodynamic limitations [14]. In other words, a lower heart rate during exercise could be considered a symptom rather than a cause of the problem. Claessen et al., using exercise cardiac magnetic resonance imaging and invasive pressure recording, found that chronotropic responsiveness was preserved in Fontan patients, which indicated normal sinoatrial function [14]. However, peak heart rate and overall exercise tolerance were impaired, a phenomenon likely secondary to diminished systemic ventricular filling, a declining stroke volume, and an early plateau in cardiac output [14]. The authors suggested that the term “chronotropic constraint” rather than “chronotropic incompetence” should be used in the case of the Fontan circulation [14]. In our study, patients with better %VO_2pred_ (group A) had significantly higher chronotropic index and lower heart rate reserve. At the same time, they used beta-blockers significantly less often than group B. This is consistent with the study by Butts et al., who found that carvedilol use in Fontan patients decreased peak heart rate without improving peak oxygen consumption and caused a mild increase in NT-proBNP [16]. Overall, chronotropic constraint—defined analogously to chronotropic incompetence (e.g., CI < 80%)—could represent a valuable prognostic marker in Fontan patients, although additional studies are required to confirm this association.

A single functional ventricle with right ventricular morphology supporting the Fontan circulation is generally considered as an unfavorable prognostic factor, particularly with regard to its potential to elevate the likelihood of developing various complications [8,17]. In a large, multicenter study by Dib et al., Fontan patients with systemic right ventricle had >2-fold higher risk of adverse cardiovascular events [8]. Although data on the long-term survival of Fontan patients with a systemic RV are currently limited, it is hypothesized that as more of these patients reach adulthood, survival trends among all Fontan patients will be significantly affected [8]. In our cohort, patients with a systemic RV exhibited a higher prevalence of ventricular arrhythmias and elevated NT-proBNP and hsTnT levels compared to those with a systemic LV. However, no statistically significant differences in exercise capacity parameters were observed between the two groups, which may be attributable to the limited sample size. Interestingly, Dhauna et al. also observed no significant differences in CPET parameters between systemic RV and LV in Fontan patients [18]. Pulmonary artery stenting was significantly more frequent in patients with a systemic RV. This finding is unsurprising, as stenosis of the left pulmonary artery remains a widely acknowledged complication secondary to the surgical palliation of hypoplastic left heart syndrome (HLHS) [19], and most patients with a systemic RV in our cohort had HLHS as their initial defect.

We found negative correlations between %VO_2pred_ and NT-proBNP, MCHC, and ferritin and positive correlations between %VO_2pred_ and both total protein and TIBC. NT-proBNP is a widely used biomarker in cardiology, and its measurement is recommended in patients with Fontan circulation [17], although robust evidence for its utility in this context is lacking. In our review of blood biomarkers in the Fontan population, we summarized the available evidence and concluded that markedly elevated NT-proBNP levels are most likely associated with Fontan failure, although additional data are needed [20]. The negative correlation between %VO_2pred_ and NT-proBNP is noteworthy; however, our analysis did not identify NT-proBNP as a predictor in the regression model. Total protein is routinely measured in Fontan patients as a screening test for protein-losing enteropathy (PLE) [17]. Low total protein levels in Fontan patients may indicate abnormal loss of serum proteins into the intestinal lumen. A diagnosis of PLE is associated with a markedly worse prognosis [17]. Ferritin and TIBC (and indirectly MCHC) are parameters associated with iron metabolism [21]. Although iron deficiency is a remarkably common finding among individuals with Fontan physiology [22], we did not identify this disorder in our cohort, and the observed correlations remain inconclusive.

In our cohort, higher maximal diastolic blood pressure during CPET was significantly and independently associated with %VO_2pred_. Diastolic blood pressure is primarily influenced by cardiac output and the resistance of peripheral blood vessels. When exercising, cardiac output rises while peripheral vascular resistance drops due to the vasodilation of small vessels in the active skeletal muscles [23]. Physiologically, as exercise progresses, diastolic blood pressure remains almost unchanged as a result of vasodilatation and a decrease of total peripheral resistance [24]. The DBP response to exercise in the Fontan circulation has not been thoroughly studied. The median resting DBP in our cohort was 70 mmHg (IQR 60–80), and the median maximal DBP [DBP_max_] was 80 mmHg (IQR 80–90). In a study by Egbe et al., which included 323 patients with Fontan circulation, mean resting DBP was 73 ± 13 mmHg, and mean DBP at peak exercise was 66 ± 11 mmHg [25].

In our cohort, lower concentration of high-sensitivity troponin T was identified as an independent predictor of %VO_2pred_. There is some evidence of hs-TnT being a prognostic biomarker in ACHD. Elevated hs-TnT signifies ongoing subclinical damage to cardiomyocytes, an effect that can arise not only from ischemia but also from myocardial wall strain or underlying inflammation [26]. Regarding the Fontan circulation, in our review on blood biomarkers in this population, we summarized the available data and concluded that there is some evidence suggesting that elevated hs-TnT may identify Fontan patients at higher risk of cardiovascular events, but further studies are needed [20]. Our current findings add to the evidence supporting the potential use of hs-TnT as a biomarker in the Fontan circulation.

This study has several limitations. Its retrospective design may have introduced bias and unmeasured confounders. The relatively small and heterogeneous cohort limits statistical power and generalizability. As all patients were treated at a single center and were of the same race and ethnicity, the findings may not be applicable to broader or more diverse populations.

## 5. Conclusions

In conclusion, in this retrospective cohort study of 50 adults with Fontan circulation, we identified higher chronotropic index, higher maximal diastolic blood pressure during CPET, and lower serum concentrations of high-sensitivity troponin T (hsTnT) as independent predictors of %VO_2pred_ in our cohort. These findings suggest that the absence of chronotropic incompetence (or rather “chronotropic constraint”) is a positive predictor of exercise capacity. Moreover, our results add to the evidence that hsTnT is a potential biomarker in this population. Further studies are warranted to validate these parameters for clinical assessment and risk stratification in Fontan patients.

## Figures and Tables

**Table 1:** Clinical characteristics of the study cohort according to the mean %VO_2pred_.

	All Patients [n = 50]	Group A (%VO_2pred_ > 62.3%) [n = 25]	Group B (%VO_2pred_ ≤ 62.3%) [n = 25]	*p*
Age [years]	22 (IQR 20–24)	23 (IQR 21–24)	21 (IQR 20–23)	0.06
Gender (female) [n, %]	25 (50%)	**16 (64%)**	**9 (36%)**	**0.047**
BMI [kg/m^2^]	21.58 (IQR 19.16–23.71)	21.38 (IQR 18.83–23.71)	21.6 (IQR 19.23–23.5)	0.64
Resting SpO_2_ [%]	92 (IQR 91–96)	**94 (IQR 92–96)**	**92 (IQR 91–95)**	**0.03**
Age at Fontan [months]	69.5 ± 29	76 ± 30	63 ± 27	0.1
Systemic LV [n, %]	23 (46%)	14 (56%)	9 (36%)	0.16
FALD [n, %]	22 (44%)	11 (44%)	11 (44%)	-
Use of beta-blockers [n, %]	19 (38%)	**4 (16%)**	**15 (60%)**	**0.003**
Use of ACE-I [n, %]	39 (78%)	18 (72%)	21 (84%)	0.31
Use of MRA [n, %]	34 (68%)	14 (56%)	20 (80%)	0.69
Pulmonary artery stenting [n, %]	21 (42%)	8 (32%)	13 (52%)	0.15
Fontan tunnel stenting [n, %]	16 (32%)	6 (24%)	10 (40%)	0.23
Systemic ventricle wall motion abnormalities [n, %]	11 (22%)	5 (10%)	6 (12%)	0.73
SVC-RPA max flow [cm/s]	37.72 ± 8.07	37.84 ± 7.06	37.6 ± 9.12	0.91
IVC-RPA max flow [cm/s]	41.5 (IQR 32–53)	43 (IQR 35–53)	41 (IQR 30–52)	0.31
Average HR in 24 h Holter ECG [beats/min]	71 ± 9	71 ± 8	71 ± 10	0.86
ExSV [n]	10 (IQR 1–30)	10 (IQR 0–20)	10 (IQR 3–45)	0.43
ExV [n]	5 (IQR 0–128)	**2 (IQR 0–17)**	**35 (IQR 1–440)**	**0.03**
Mean SBP in ABMP [mmHg]	113 ± 10.5	115 ± 11	111 ± 9	0.2
Mean DBP in ABPM [mmHg]	67 ± 5.5	**68 ± 5**	**65 ± 5**	**0.047**
Hb [g/dL]	15.3 (IQR 14,3–16)	15.1 (IQR 12.6–15.7)	15.4 (IQR 14.8–16.2)	0.18
MCHC [g/dL]	34.5 (IQR 33.8–35.5)	**34.1 (IQR 33.7–34.7)**	**35 (IQR 34.2–35.8)**	**0.02**
TIBC [µg/dL]	335.5 (IQR 285–360)	**352 (IQR 319–367)**	**319 (IQR 283–347)**	**0.043**
Fe [µg/dL]	91 (IQR 63–117)	97 (IQR 77–135)	86 (IQR 57–107)	0.1
Ferritin [ng/mL]	97.55 (40–164.2)	85.2 (IQR 35.9–112)	114 (IQR 56.9–171.6	0.1
APTT [s]	32.3 (IQR 29.3–35.3)	32.7 (IQR 29.3–35.3)	31.9 (29.3–34.9)	0.59
TSH [uIU/mL]	2.69 (IQR 2–3.9)	2.68 (IQR 2.01–3.46)	3.31 (2–3.94)	0.65
Vitamin D [ng/mL]	24.05 (IQR 18.9–32.6)	25 (IQR 18.7–34.1)	22.7 (IQR 20.8–27.7)	0.49
NT-proBNP [pg/mL]	108 (IQR 44–207)	83 (IQR 37.8–145)	138 (IQR 53–295)	0.17
hsTnT [pg/mL]	5.1 (IQR 3.8–6.2)	**4.7 (IQR 3.6–5.5)**	**5.7 (IQR 4.4–6.7)**	**0.03**
Time of exercise during CPET [s]	605 (IQR 455–711)	**629 (IQR 545–727)**	**596 (IQR 410–632)**	**0.016**
MET achieved	11.6 (IQR 10–13.5)	**13.5 (IQR 10.2–13.7)**	**10.7 (IQR 7.7–13.4)**	**0.008**
SBP_rest_ [mmHg]	110 (IQR 100–120)	110 (IQR 100–120)	110 (IQR 100–120)	0.19
DBP_rest_ [mmHg]	70 (IQR 60–80)	70 (IQR 70–80)	70 (IQR 60–80)	0.57
SBP_max_ [mmHg]	140 (IQR 140–160)	150 (IQR 140–170)	140 (IQR 140–160)	0.11
DBP_max_ [mmHg]	**80 (IQR 80–90)**	**80 (IQR 80–90)**	**80 (IQR 70–90)**	**0.03**
HR_peak_ [beats/min]	171 (IQR 142–180)	**176 (IQR 171–184)**	**151 (IQR 134–173)**	**0.007**
CI [%]	75.35 (IQR 49.23–83.33)	**77.78 (IQR 75–87.5)**	**60.63 (IQR 47.24–78.45)**	**0.007**
HRr	26.5 (IQR 17–52)	**20 (IQR 12–30)**	**50 (IQR 25–65)**	**0.004**
O_2_pulse [mL/beat]	10 (IQR 8–11.8)	10 (IQR 8–12)	10 (IQR 8–11)	0.47
VE_peak_ [L/min]	61.35 (IQR 51.7–79.9)	**68.3 (IQR 54.8–94.7)**	**59.9 (IQR 47.2–71)**	**0.018**
%VO_2_-AT [%]	47 (IQR 34–53)	**50 (IQR 44–56)**	**43 (IQR 33–49)**	**0.03**
RER	1.087 ± 0.06	1.097 ± 0.05	1.075 ± 0.06	0.17
VE/VCO_2_slope	32.85 (IQR 30.7–38.4)	32.85 (IQR 29.8–37.7)	32.85 (IQR 32–38.6)	0.66

Abbreviations: %VO_2_-AT—oxygen uptake at anaerobic threshold; %VO_2pred_—percent achieved of predicted peak exercise oxygen uptake; ABMP—ambulatory blood pressure monitoring; ACE-I—angiotensin-converting enzyme inhibitor; APTT—activated partial thromboplastin time; BMI—body mass index; CI—chronotropic index; CPET—cardiopulmonary exercise testing; DBP—diastolic blood pressure; DBP_max_—maximal diastolic blood pressure during CPET; DBP_rest_—resting diastolic blood pressure; ECG—electrocardiography; ExSV—supraventricular extrasystolic beats; ExV—ventricular extrasystolic beats; FALD—Fontan-associated liver disease; Fe—serum ferrum concentration; Hb—hemoglobin concentration; HR—heart rate; HR_peak_—heart rate at peak exercise; HRr—heart rate reserve; hsTnT—high-sensitivity troponin T; IQR—interquartile range; IVC—inferior vena cava; LV—left ventricle; MCHC—mean corpuscular hemoglobin concentration; MET—metabolic equivalent of task; MRA—mineralocorticoid receptor antagonist; NT-proBNP—N-terminal prohormone of brain natriuretic peptide; O_2_pulse—oxygen pulse; RER—respiratory exchange ratio; RPA—right pulmonary artery; SBP_max_—maximal systolic blood pressure during CPET; SBP_rest_—resting systolic blood pressure; SpO_2_—oxygen saturation; SVC—superior vena cava; TIBC—total iron-binding capacity; TSH—thyroid-stimulating hormone; VE/VCO_2_slope—minute ventilation/carbon dioxide production slope; VE_peak_—ventilation at peak exercise.

**Table 2:** Clinical characteristics of the study cohort according to the systemic ventricle.

	All Patients [n = 50]	Systemic LV [n = 23]	Systemic RV [n = 23]	*p*
Age [years]	22 (IQR 20–24)	22 (IQR 20–25)	21 (IQR 19–23)	0.12
Gender (female) [n, %]	25 (50%)	14 (61%)	8 (35%)	0.08
BMI [kg/m^2^]	21.6 (IQR 19.2–23.7)	22.1 (IQR 20.2–24.5)	19.92 (IQR 18.04–22.52)	0.08
Resting SpO_2_ [%]	92 (IQR 91–96)	92 (IQR 91–96)	93 (IQR 92–96)	0.56
Age at Fontan [months]	70 ± 29	75.5 ± 32	62 ± 24	0.12
FALD [n, %]	22 (44%)	9 (39%)	12 (52%)	0.37
Use of beta-blockers [n, %]	19 (38%)	10 (43%)	8 (35%)	0.55
Use of ACE-I [n, %]	39 (78%)	**14 (61%)**	**22 (96%)**	**0.01**
Use of MRA [n, %]	34 (68%)	**11 (48%)**	**21 (91%)**	**0.003**
Pulmonary artery stenting [n, %]	21 (42%)	**4 (17%)**	**16 (70%)**	**0.0008**
Fontan tunnel stenting [n, %]	16 (32%)	10 (43%)	6 (26%)	0.22
Systemic ventricle wall motion abnormalities [n, %]	11 (22%)	3 (13%)	7 (30%)	0.28
SVC-RPA max flow [cm/s]	37 (IQR 32–41)	37 (IQR 32–41)	37 (IQR 30–45)	0.99
IVC-RPA max flow [cm/s]	41.5 (IQR 32–53)	41 (IQR 29–47)	45 (IQR 32–55)	0.19
Average HR in 24 h Holter ECG [beats/min]	71 ± 9	71 ± 8	71 ± 11	0.88
ExSV [n]	10 (IQR 1–30)	16 (IQR 3–167)	5 (IQR 0–30)	0.1
ExV [n]	5 (IQR 0–128)	**2 (IQR 0–39)**	**33 (IQR 2–440)**	**0.04**
Mean SBP in ABPM [mmHg]	113 ± 10.5	112 ± 11	114 ± 10.5	0.45
Mean DBP in ABPM [mmHg]	67 ± 5.5	67 ± 11	67 ± 10.5	0.9
MCHC [g/dL]	34.5 (IQR 33.8–35.5)	34.3 (IQR 33.7–35.6)	34.7 (IQR 34.2–35)	0.92
TIBC [µg/dL]	335.5 (IQR 285–360)	337 (IQR 285–367)	333 (IQR 281–360)	0.61
APTT [s]	32.3 ± 4.3	31.14 ± 3.34	34.1 ± 4.89	0.02
TSH [uIU/mL]	2.69 (IQR 2–3.9)	**2.39 (IQR 1.74–3.19)**	**3.62 (IQR 2.04–5.37)**	**0.03**
Vitamin D [ng/mL]	24.05 (IQR 18.9–32.6)	**21.6 (IQR 17.8–31.3)**	**28.5 (IQR 22.3–35.12)**	**0.03**
NT-proBNP [pg/mL]	108 (IQR 44–207)	**65 (IQR 26–145)**	**139 (IQR 54–391)**	**0.04**
hsTnT [pg/mL]	5.1 (IQR 3.8–6.2)	**4.7 (IQR 3.4–5.5)**	**5.7 (IQR 4.4–7.3)**	**0.03**
Time of exercise during CPET [s]	605 (IQR 455–711)	561 (IQR 436–712)	609 (IQR 569–723)	0.4
MET achieved	11.7 ± 3.4	11.2 ± 3.3	12.47 ± 3.3	0.21
SBP_rest_ [mmHg]	110 (IQR 100–120)	110 (IQR 100–120)	110 (IQR 100–120)	0.98
DBP_rest_ [mmHg]	70 (IQR 60–80)	70 (IQR 60–80)	70 (IQR 60–80)	0.88
SBP_max_ [mmHg]	140 (IQR 140–160)	140 (IQR 140–160)	150 (IQR 130–160)	>0.99
DBP_max_ [mmHg]	80 (IQR 80–90)	80 (IQR 80–90)	80 (IQR 80–90)	0.51
HR_peak_ [beats/min]	171 (IQR 142–180)	173 (IQR 139–184)	165 (IQR 142–179)	0.39
CI [%]	75.4 (IQR 49.2–83.3)	77.8 (IQR 48.7–89)	64.3 (IQR 49.2–78.4)	0.25
HRr	26,5 (IQR 17–52)	22 (IQR 10–60)	33 (IQR 19–52)	0.36
O_2_pulse [mL/beat]	10 (IQR 8–11.8)	10 (IQR 8–12)	10 (IQR 8–12)	0.95
VE_peak_ [L/min]	67 ± 24.3	65.1 ±25.2	68.9 ± 24.9	0.61
VO_2_/kg [mL/kg/min]	24.9 ± 5.9	24.7 ± 6.2	25.6 ± 5.6	0.59
%VO_2pred_ [%]	63.2 ± 11.9	66.09 ± 11.96	60.35 ± 12.19	0.11
%VO_2_-AT [%]	47 (IQR 34–53)	49 (IQR 33–55)	47 (IQR 37–53)	0.9
RER	1.087 ± 0.06	1.093 ± 0.05	1.082 ± 0.06	0.5
VE/VCO_2_slope	32.9 (IQR 30.7–38.4)	32.9 (IQR 31–36.6)	32.9 (IQR 29.8–40)	0.6

Abbreviations: %VO_2_-AT—oxygen uptake at anaerobic threshold; %VO_2pred_—percent achieved of predicted peak exercise oxygen uptake; ACE-I—angiotensin-converting enzyme inhibitor; APTT—activated partial thromboplastin time; BMI—body mass index; CI—chronotropic index; CPET—cardiopulmonary exercise testing; DBP—diastolic blood pressure; DBP_max_—maximal diastolic blood pressure during CPET; DBP_rest_—resting diastolic blood pressure; ECG—electrocardiography; ExSV—supraventricular extrasystolic beats; ExV—ventricular extrasystolic beats; FALD—Fontan-associated liver disease; HR—heart rate; HR_peak_—heart rate at peak exercise; HRr—heart rate reserve; hsTnT—high-sensitivity troponin T; IQR—interquartile range; IVC—inferior vena cava; LV—left ventricle; MCHC—mean corpuscular hemoglobin concentration; MET—metabolic equivalent of task; MRA—mineralocorticoid receptor antagonist; NT-proBNP—N-terminal prohormone of brain natriuretic peptide; O_2_pulse—oxygen pulse; RER—respiratory exchange ratio; RPA—right pulmonary artery; RV—right ventricle; SBP_max_—maximal systolic blood pressure during CPET; SBP_rest_—resting systolic blood pressure; SpO_2_—oxygen saturation; SVC—superior vena cava; TIBC—total iron-binding capacity; TSH—thyroid-stimulating hormone; VE/VCO_2slope_—minute ventilation/carbon dioxide production slope; VE_peak_—ventilation at peak exercise.

**Table 3:** Univariable and multivariable linear regression analysis for %VO_2pred_.

Variable	Univariate Analysis			Multivariate Analysis		
	Coefficient	95%CI	*p*	Coefficient	95%CI	*p*
Gender (female)	0.43	(−0.13)–1.00	0.13	-	-	-
Resting SpO_2_	0.21	(−0.07)–0.49	0.14	-	-	-
Use of beta-blockers	−0.8	(−1.35)–(−0.25)	**0.005**	-	-	-
ExV	−0.12	(−0.41)–0.16	0.39	-	-	-
MCHC	−0.11	(−0.39)–0.18	0.46	-	-	-
TIBC	0.31	0.03–0.58	0.03	-	-	-
hsTnT	−0.35	(−0.62)–(−0.08)	**0.01**	**−0.31**	**(−0.539)–(−0.090)**	**0.007**
NT-proBNP	−0.32	(−0.6)–(−0.05)	**0.023**	-	-	-
HR_peak_	0.39	0.13–0.66	**0.005**	-	-	-
Chronotropic index	0.43	0.17–0.7	**0.0017**	**0.31**	**0.081–0.542**	**0.009**
HRr	−0.41	(−0.67)–(−0.14)	**0.004**	-	-	-
VE_peak_	0.36	0.09–0.63	**0.01**	-	-	-
%VO_2_-AT	0.3	0.02–0.58	**0.035**	-	-	-
DBP_max_	0.47	0.22–0.73	**0.0006**	**0.4**	**0.168–0.627**	**0.001**

Abbreviations: %VO_2_-AT—oxygen uptake at anaerobic threshold; %VO_2pred_—percent achieved of predicted peak exercise oxygen uptake; 95%CI—95% confidence interval; DBP_max_—maximal diastolic blood pressure during CPET; ExV—ventricular extrasystolic beats; HR_peak_—heart rate at peak exercise; HRr—heart rate reserve; hsTnT—high-sensitivity troponin T; MCHC—mean corpuscular hemoglobin concentration; NT-proBNP—N-terminal prohormone of brain natriuretic peptide; SpO_2_—oxygen saturation; TIBC—total iron-binding capacity; VE_peak_—ventilation at peak exercise.

## Data Availability

The data that support the findings of this study are available from the corresponding author, A.W., upon reasonable request.

## References

[1] Baumgartner H., De Backer J., Babu-Narayan S.V., Budts W., Chessa M., Diller G.-P., Lung B., Kluin J., Lang I.M., Meijboom F. (2021). 2020 ESC Guidelines for the Management of Adult Congenital Heart Disease. Eur. Heart J..

[2] Wittczak A., Dryżek P., Maciejewski M., Kula-Mazurek A., Moszura T., Bikiewicz A., Bielecka-Dabrowa A. (2023). Successful Complex Percutaneous Intervention in Patient with Fontan Circulation and Severe Heart Failure: A Case Report. Clin. Case Rep..

[3] Leczycki P., Banach M., Maciejewski M., Bielecka-Dabrowa A. (2022). Heart Failure Risk Predictions and Prognostic Factors in Adults with Congenital Heart Diseases. Front. Cardiovasc. Med..

[4] Smarż K., Jaxa-Chamiec T., Chwyczko T., Główczyńska R., Jegier A., Niedoszytko P., Piotrowicz E., Rybicki J., Straburzyńska-Migaj E., Szalewska D. (2019). Cardiopulmonary Exercise Testing in Adult Cardiology: Expert Opinion of the Working Group of Cardiac Rehabilitation and Exercise Physiology of the Polish Cardiac Society. Kardiol. Pol..

[5] Guazzi M., Adams V., Conraads V., Halle M., Mezzani A., Vanhees L., Arena R., Fletcher G.F., Forman D.E., Kitzman D.W. (2012). Clinical Recommendations for Cardiopulmonary Exercise Testing Data Assessment in Specific Patient Populations. Circulation.

[6] Tran D.L., Celermajer D.S., Ayer J., Grigg L., Clendenning C., Hornung T., Justo R., Davis G.M., d’Udekem Y., Cordina R. (2021). The “Super-Fontan” Phenotype: Characterizing Factors Associated With High Physical Performance. Front. Cardiovasc. Med..

[7] Paridon S.M., Mitchell P.D., Colan S.D., Williams R.V., Blaufox A., Li J.S., Margossian R., Mital S., Russell J., Rhodes J. (2008). A Cross-Sectional Study of Exercise Performance during the First 2 Decades of Life after the Fontan Operation. J. Am. Coll. Cardiol..

[8] Dib N., Chaix M.-A., Samuel M., Hermann Honfo S., Hamilton R.M., Aboulhosn J., Broberg C.S., Cohen S., Cook S., Dore A. (2024). Cardiovascular Outcomes in Fontan Patients with Right vs Left Univentricular Morphology: A Multicenter Study. JACC Adv..

[9] Wittczak A., Kobierecki M., Banach M., Bielecka-Dabrowa A. (2025). Predictors of Higher Peak Exercise Oxygen Uptake in a Cohort of Adult Patients with the Fontan Circulation. Eur. Heart J..

[10] Society P.C. (2025). The 29th International Congress of the Polish Cardiac Society. Pol. Heart J. (Kardiol. Pol.).

[11] Gewillig M., Mertens L.L. (2021). Echocardiographic Assessment of Functional Single Ventricles after the Fontan Operation. Echocardiography in Pediatric and Congenital Heart Disease.

[12] Dobre D., Zannad F., Keteyian S.J., Stevens S.R., Rossignol P., Kitzman D.W., Landzberg J., Howlett J., Kraus W.E., Ellis S.J. (2013). Association between Resting Heart Rate, Chronotropic Index, and Long-Term Outcomes in Patients with Heart Failure Receiving β-Blocker Therapy: Data from the HF-ACTION Trial. Eur. Heart J..

[13] Zweerink A., van der Lingen A.-L.C., Handoko M.L., van Rossum A.C., Allaart C.P. (2018). Chronotropic Incompetence in Chronic Heart Failure. Circ. Heart Fail..

[14] Claessen G., La Gerche A., Van De Bruaene A., Claeys M., Willems R., Dymarkowski S., Bogaert J., Claus P., Budts W., Heidbuchel H. (2019). Heart Rate Reserve in Fontan Patients: Chronotropic Incompetence or Hemodynamic Limitation?. J. Am. Heart Assoc..

[15] Okólska M., Skubera M., Matusik P., Płazak W., Pająk J., Róg B., Podolec P., Tomkiewicz-Pająk L. (2021). Chronotropic Incompetence Causes Multiple Organ Complications in Adults after the Fontan Procedure. Pol. Heart J. (Kardiol. Pol.).

[16] Butts R., Atz A.M., BaezHernandez N., Sutcliffe D., Reisch J., Mahony L. (2021). Carvedilol Does Not Improve Exercise Performance in Fontan Patients: Results of a Crossover Trial. Pediatr. Cardiol..

[17] Rychik J., Atz A.M., Celermajer D.S., Deal B.J., Gatzoulis M.A., Gewillig M.H., Hsia T.-Y., Hsu D.T., Kovacs A.H., McCrindle B.W. (2019). Evaluation and Management of the Child and Adult with Fontan Circulation: A Scientific Statement from the American Heart Association. Circulation.

[18] Dhauna J., Aboulhosn J., Lluri G. (2022). Cardiopulmonary Exercise Test Outcomes in Fontan Patients with Right Versus Left Single Ventricle Morphology. World J. Pediatr. Congenit. Heart Surg..

[19] Noonan P., Kudumula V., Anderson B., Ramchandani B., Miller P., Dhillon R., Mehta C., Stumper O. (2016). Stenting of the Left Pulmonary Artery after Palliation of Hypoplastic Left Heart Syndrome. Catheter. Cardiovasc. Interv..

[20] Wittczak A., Mazurek-Kula A., Banach M., Piotrowski G., Bielecka-Dabrowa A. (2025). Blood Biomarkers as a Non-Invasive Method for the Assessment of the State of the Fontan Circulation. J. Clin. Med..

[21] Rusch J.A., van der Westhuizen D.J., Gill R.S., Louw V.J. (2023). Diagnosing Iron Deficiency: Controversies and Novel Metrics. Best Pract. Res. Clin. Anaesthesiol..

[22] van Hassel G., Rivrud S.C.S., Timmerman F.J., van der Meer P., Hoendermis E.S., Liem E.T., Berger R.M.F., van Melle J.P. (2024). Iron Deficiency in Patients with a Fontan Circulation and Its Impact on Exercise Capacity. Open Heart.

[23] Brett S.E., Ritter J.M., Chowienczyk P.J. (2000). Diastolic Blood Pressure Changes during Exercise Positively Correlate with Serum Cholesterol and Insulin Resistance. Circulation.

[24] Laukkanen J.A., Kurl S. (2012). Blood Pressure Responses during Exercise Testing-Is up Best for Prognosis?. Ann. Med..

[25] Egbe A.C., Ali A.E., Miranda W.R., Connolly H.M., Borlaug B.A. (2025). Aerobic Capacity of Adults with Fontan Palliation: Disease-Specific Reference Values and Relationship to Outcomes. Circ. Heart Fail..

[26] Willinger L., Brudy L., Meyer M., Oberhoffer-Fritz R., Ewert P., Müller J. (2021). Prognostic Value of Non-Acute High Sensitive Troponin-T for Cardiovascular Morbidity and Mortality in Adults with Congenital Heart Disease: A Systematic Review. J. Cardiol..

